# Infective Endocarditis Masquerading as Rheumatoid Arthritis

**DOI:** 10.7759/cureus.5626

**Published:** 2019-09-11

**Authors:** Basil Peechakara, Amey Kadam, Megha Mewada, Akshay Nakrani

**Affiliations:** 1 Internal Medicine, Aastha Lifecare Hospital and Medical Centre, Mumbai, IND

**Keywords:** infective endocarditis, streptococcus gordonii, rheumatoid factor

## Abstract

Infective endocarditis (IE) is associated with high inflammatory markers including rheumatoid factor (RF). Diagnosis can be difficult when it presents with musculoskeletal symptoms, and a raised RF titer as it points towards an autoimmune joint disease. It is imperative to rule out IE by echocardiography and blood cultures. A 42-year-old male with type two diabetes mellitus presented to our hospital with severe back pain, hemoptysis, mild pain in multiple joints, and low-grade fever for three months. He was previously seen by a rheumatologist and was clinically diagnosed with rheumatoid arthritis along with a RF level of 505.3 IU/mL. After an extensive investigation, transthoracic echocardiography (TTE) showed vegetations on the ventricular side of the aortic valve. Transesophageal echocardiography (TEE) confirmed vegetations on the aortic valve and also detected anterior mitral valve leaflet perforation with regurgitation. He was treated with ceftriaxone and gentamycin for six and two weeks, respectively. High RF is associated with IE possibly due to an intense immune response generated by a chronic intravascular infection. Echocardiography should be performed in a suspected case as a prompt diagnosis is related to better outcomes.

## Introduction

Diagnosis of infective endocarditis (IE) can be challenging due to the involvement of multiple organ systems. Data on various presentations of the disease becomes important in order to update the existing clinical criteria for diagnosis. Prompt diagnosis is necessary as despite optimal medical care, mortality approaches 30% at one year [1].

## Case presentation

**Figure 1:**
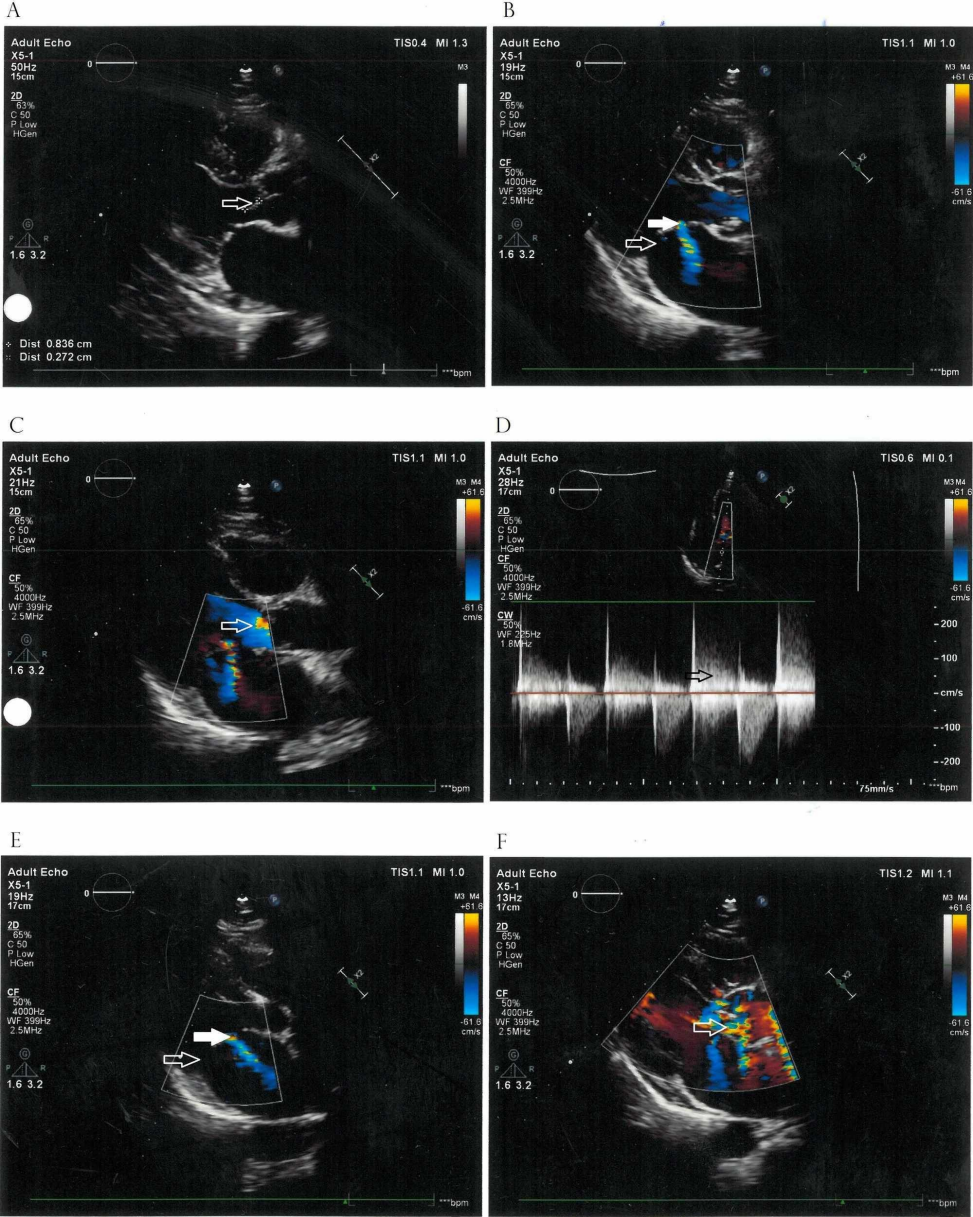
Transesophageal echocardiography showing vegetations on the aortic valve. (A) Hollow arrow shows vegetations on the ventricular side of the aortic valve. (B) Solid arrow shows regurgitation through a perforated anterior mitral leaflet. Hollow arrow shows the normal position of the blood flow through the mitral valve. (C) Hollow arrow shows aortic regurgitation. (D) Hollow arrow shows aortic regurgitation. (E) Solid arrow shows regurgitation through a perforated anterior mitral leaflet. Hollow arrow shows the normal position of the blood flow through the mitral valve. (F) Hollow arrow shows aortic regurgitation.

A 42-year-old male with type two diabetes mellitus presented to our hospital with severe back pain, hemoptysis, mild pain in multiple joints, and low-grade fever for three months. He was seen by a rheumatologist and noted to have restricted left shoulder movement, right wrist swelling, and bilateral metacarpal squeeze test positive suggestive of inflammation. He also had microcytic hypochromic anemia (hemoglobin: 9.3 g/dL) with anisocytosis and rouleaux formation, erythrocyte sedimentation rate of 109 mm, and C-reactive protein of 44.4 mg/dL. He was found to have very high rheumatoid factor (RF) titer (505.3 IU/mL) which had increased from an RF of 339.2 IU/mL two weeks back and was clinically diagnosed with rheumatoid arthritis. However, anti-cyclic citrullinated peptide (anti-ccp) and antinuclear antibody were negative. He was being treated with prednisone, methotrexate, and hydroxychloroquine. On admission, he was screened for vasculitis including proteinase 3 anti-neutrophilic cytoplasmic antibody and myeloperoxidase anti-neutrophil cytoplasmic antibody (PR3-ANCA and MPO-ANCA), serum protein electrophoresis, and complement (C3 and C4) levels. Lumbar spinal X-ray with sacroiliac joints was done to rule out ankylosing spondylitis. Workup was insignificant except for mild hypergammaglobulinemia (2.38 g/dL) without monoclonal bands. Anti-streptolysin O, malarial parasite, and widal test were negative. MRI of the spine showed disc herniation at L4-L5 and L5-S1 levels with degenerative changes. A transthoracic echocardiography (TTE) was performed to investigate for IE and it showed vegetations on the ventricular side of the noncusp leaflets of the aortic valve associated with moderate aortic regurgitation. Prednisone, methotrexate, and hydroxychloroquine were subsequently discontinued. Later blood cultures were positive for *Streptococcus gordonii* (part of the group *S. viridans*), classified as a typical microbe in IE. Transesophageal echocardiography (TEE) confirmed vegetations on the aortic valve and also detected anterior mitral valve leaflet perforation with regurgitation (as shown in Figure 1A). Positron emission tomography (PET) scan showed areas of metabolic activity in L4 and L5 end plates. Antibiotic sensitivity showed sensitivity to ceftriaxone and gentamycin, which were continued after discharge for six and two weeks, respectively, with weekly blood cultures.

## Discussion

A study by Gouriet et al. showed that RF was elevated in 36% of the definite cases and 19% of the rejected cases. Among the other markers tested like C-reactive protein, erythrocyte sedimentation rate and tumor necrosis factor, only RF had a statistically significant difference [2]. It is theorized that the development of RF could be related to an intense immune response generated by a chronic intravascular infection.

Therefore, musculoskeletal complaints and the prevalence of positive RF in patients with fever of unknown origin makes it challenging as it points the diagnosis towards an autoimmune multiple joint disease. It is imperative to screen for IE with blood cultures and echocardiography. TTE has a sensitivity and specificity of 61% and 94% when compared to TEE as the imaging gold standard [3]. Duke’s criterion is the gold standard for diagnosis but the heterogeneity of the patient presentations necessitates clinical judgement in addition to applying the criterion [4].

Infective endocarditis requires six weeks of antibiotic treatment. It is noteworthy that in a review by Dadon et al, two out of 11 patients with IE by *S. gordonii* presented with back pain, and both required an additional six weeks of antibiotic therapy in addition to the standard six week therapy [5].

## Conclusions

Infective endocarditis is associated with high inflammatory markers including RF. Diagnosis can be difficult when it presents with musculoskeletal symptoms and a raised RF titer as it points towards an autoimmune joint disease. It is imperative to rule out IE by echocardiography and blood cultures.

## References

[1] Cahill TJ, Baddour LM, Habib G (2017). Challenges in infective endocarditis. J Am Coll Cardiol.

[2] Gouriet F, Bothelo-Nevers E, Coulibaly B, Raoult D (2006). Evaluation of sedimentation rate, rheumatoid factor, C-reactive protein, and tumor necrosis factor for the diagnosis of infective endocarditis. Clin Vaccine Immunol.

[3] Bai AD, Steinberg M, Showler A (2017). Diagnostic accuracy of transthoracic echocardiogmraphy for infective endocarditis findings using transesophageal echocardiography as the reference standard: a meta-analysis. J Am Soc Echocardiogr.

[4] Holland TL, Baddour LM, Bayer AS, Hoen B, Miro JM, Fowler Jr. VG (2016). Infective endocarditis. Nat Rev Dis Primers.

[5] Dadon Z, Cohen A, Szterenlicht YM (2017). Spondylodiskitis and endocarditis due to Streptococcus gordonii. Ann Clin Microbiol Antimicrob.

